# Toxicity Assessment of the Binary Mixtures of Aquatic Organisms Based on Different Hypothetical Descriptors

**DOI:** 10.3390/molecules27196389

**Published:** 2022-09-27

**Authors:** Meng Ji, Lihong Zhang, Xuming Zhuang, Chunyuan Tian, Feng Luan, Maria Natália D. S. Cordeiro

**Affiliations:** 1 College of Chemistry and Chemical Engineering, Yantai University, Yantai 264005, China; 2 LAQV@REQUIMTE/Department of Chemistry and Biochemistry, Faculty of Sciences, University of Porto, 4169-007 Porto, Portugal

**Keywords:** toxicity assessment, mixture, quantitative structure-activity relationships (QSAR), forward stepwise multiple linear regression (MLR), radial basis function neural networks (RBFNN)

## Abstract

Modern industrialization has led to the creation of a wide range of organic chemicals, especially in the form of multicomponent mixtures, thus making the evaluation of environmental pollution more difficult by normal methods. In this paper, we attempt to use forward stepwise multiple linear regression (MLR) and nonlinear radial basis function neural networks (RBFNN) to establish quantitative structure–activity relationship models (QSARs) to predict the toxicity of 79 binary mixtures of aquatic organisms using different hypothetical descriptors. To search for the proper mixture descriptors, 11 mixture rules were performed and tested based on preliminary modeling results. The statistical parameters of the best derived MLR model were N_train_ = 62, R^2^ = 0.727, RMS = 0.494, F = 159.537, Q^2^_LOO_ = 0.727, and Q^2^_pred_ = 0.725 for the training set; and N_test_ = 17, R^2^ = 0.721, RMS = 0.508, F = 38.773, and q^2^_ext_ = 0.720 for the external test set. The RBFNN model gave the following statistical results: N_train_ = 62, R^2^ = 0.956, RMS = 0.199, F = 1279.919, Q^2^_LOO_ = 0.955, and Q^2^_pred_ = 0.855 for the training set; and N_test_ = 17, R^2^ = 0.880, RMS = 0.367, F = 110.980, and q^2^_ext_ = 0.853 for the external test set. The quality of the models was assessed by validating the relevant parameters, and the final results showed that the developed models are predictive and can be used for the toxicity prediction of binary mixtures within their applicability domain.

## 1. Introduction

It has been widely recognized that toxic chemicals in the environment do not exist individually but are mixtures of each other; thus, research on both single and mixed toxic compounds is important [1,2]. Today, water system pollution is worthy of attention. With modern industrial development, various water bodies suffer from damage due to industrial wastewater discharge and the discharge of pesticides, organic chemicals, and other pollutants from all walks of life, with the destruction of water bodies posing a great threat to the ecological environment and human health [3,4]. In the aquatic world, water pollutants hardly ever exist as a compound alone but rather contaminate water bodies as a mixture. Under existing human risk assessment regulations, the toxicity performance of toxic chemicals is simply assessed by information on the toxicity of their individual compounds, whereas the fact is that the toxicity performance of an individual compound is vastly divergent from that of a mixture and that interactions between the components of a mixture can lead to synergistic and antagonistic reactions of an individual compound, resulting in significant changes in the toxicity performance of a mixture compared to a single one [5]. To address these issues, a risk assessment of the toxicity of mixtures seems to be greatly important and thus necessary.

For the combined effects of mixture toxicity evaluation, one can usually resort to two types of models: concentration addition (CA) and independent action (IA) models. As we know, the conventional CA is the most common additive toxicity model, which is based on the assumption that although the components of a mixture have the same mechanism of action with the same target on the basis, the components do not interact with each other and the rule has been highly endorsed by the US National Environmental Protection Agency and the European Commission [6,7,8]. The IA model, also termed the reaction-additive approach, assumes that the mechanisms and targets of action vary widely among the components of the mixture [9,10]. The two traditional models have their own limitations, although several new approaches based on the CA model as well as the IA model have been developed to overcome this shortcoming, e.g., the generalized concentration addition (GCA) model. CA, IA, and their optimization models also have a flawed side; they should only be used when there are no interactions between the mixture components and the mechanism of action of each component is known [11].

Currently, more methods are commonly employed to evaluate the toxicity of mixtures. Quantitative structure–activity relationship (QSAR) modeling is one of the methods used to predict the toxicity of environmental contaminants and is applied to the risk assessment of mixtures in various research fields, such as physical chemistry, medicinal chemistry, and toxicology [12]. As a computational method, the primary purpose of this method involves the use of data statistics, analysis, and other mathematical methods to correlate the activity, properties, and toxicity of a compound with its structure [13].As a theoretical study, which uses a relatively small number of compounds to establish mathematical relationships for predicting the properties of unknown compounds that fit the relationship, it reduces the burden of experimental studies and provides an alternative to animal studies [14]. The establishment of stable and reliable quantitative structure–activity relationship models relies on the calculation and selection of molecular descriptors. Thus, choosing the appropriate mixing rules of the mixing descriptors used in the models is particularly important for model quality owing to the complexity of the mechanisms of toxicity present [15].

QSAR methods have been used to evaluate the damage to aquatic ecosystems caused by aquatic toxicants. For instance, in the article by Yaqian Wang, seven phenolic and four aliphatic phenolic derivatives, including 2,4,6-trihalophenol, 2,6-dilhalogenated-4-nitrophenol, etc., in wastewater were studied by these methods [16]. In the work by Stefano Cassani et al., QSAR models based on forward stepwise multiple linear regression (MLR), partial least Squares regression (PLS), and associative neural network (ASNN) methods were developed for triazoles and benzotriazoles, and the mixture toxicity between them was predicted and analyzed [17]. In turn, the mixture effects of drug molecules on aquatic ecosystems were studied by Kabiruddin Khan et al. [18]. An evaluation of the toxic substances of interest, especially for the mixtures, is somewhat more useful.

**Figure 1:**
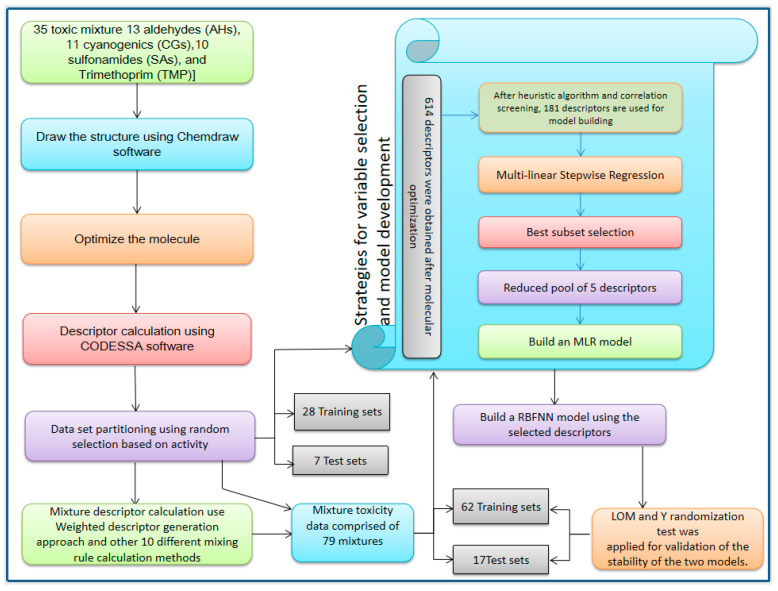
Schematic diagram of the entire approach involved in the development of the QSAR models.

The aim of this study was to develop stable QSAR models that can be used to predict the toxicity of aquatic binary mixtures at the EC50 level. The models were developed by a regression-based MLR modeling technique and a nonlinear-based radial basis function neural networks (RBFNNs) modeling technique. In our work, to obtain the proper mixture descriptors, multiple parameter combinations were used for preliminary modeling. Not only were the parameter combinations considered as a simple CA, but additional 10 mixing rules were chosen for modeling comparisons. Finally, the best combination of parameters was selected based on preliminary modeling results. A schematic diagram of the entire approach is presented in Figure 1,and more details about the methods can be found in Section 3). 

## 2. Results

### 2.1. Model Development for Individual Compounds

As shown in Figure 1, in the current study, 35 compounds present in the aqueous environment were used for model development. First, a total of 614 descriptors were obtained after molecular optimization. Then, 348 nonconforming descriptors were eliminated after the CODESSA software heuristic method (HM), and 85 descriptors were pruned out of the descriptor pool after descriptor relevance screening. Finally, 181 descriptors were left to build the model. Through the forward stepwise multiple regression method, the five representative descriptors were selected for the construction of both the MLR and the RFBNNs models. (The total and the eliminated descriptor in this study are present in the supplementary material.) To evaluate the models, the leave-out many cross-validation (LOM) and Y randomization test methods were performed. For the individual groups, the results are listed in Table 1.

As seen from the table, five descriptors were chosen for the construction of the relationship through a forward stepwise multiple linear regression approach: (1) minimum atomic state energy for a C atom (Min-C); (2) relative number of N atoms (Rn-N); (3) total point charge component of the molecular dipole (Tot-pc); (4) maximum e-n attraction for a C–H bond (Max- C-H); and (5) HOMO energy (HOMO). It is expressed by Equation (1):

pEC50 = 155.63−1.2453 × [Min-C]−7.7850 × [Rn-N] + 0.27723 × [Tot-pc]-0.33616 × [Max-C-H] + 0.23028 × [HOMO](1)

**Figure 2:**
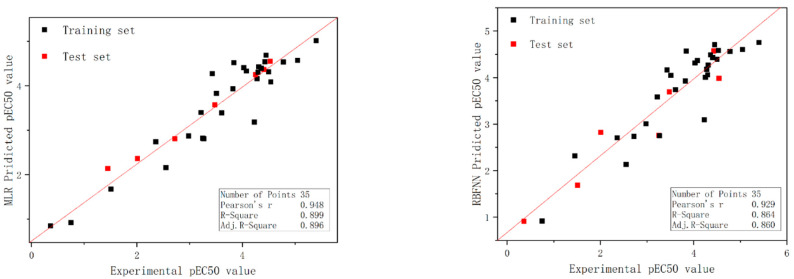
Plot of the predicted versus experimental log (EC50), including the training and test sets, from the MLR model and RBFNN model.

N_train_ = 28, R^2^ = 0.887, RMS = 0.398, F = 204.660, Q^2^_LOO_ = 0.887, and R^2^_pred_ = 0.938; and N_test_ = 7, R^2^ = 0.987, RMS = 0.297, F = 374.332, and q^2^_ext_ =0.933. The statistical results reveal that the model has excellent statistical reliability for the internal training set and outstanding predictive power for the external test set (the minimum accepted value for Q^2^_LOO_, R^2^_pred_, and q^2^_ext_ is 0.5, and for R^2^ is 0.6; in addition, the smaller the RMS value, the larger the F value, and the higher the quality of the model). In Table 2, the predicted pEC50 values, experimental pEC50 values, residual values, and individual compound names for an individual compound by each of the two models are shown, along with figures depicting the experimental and predicted value curves for the training and test sets under each of the two models in Figure 2.

An assessment of the correlation between the individual descriptors is necessary for binary mixture descriptor generation, and when the pairwise correlation between two individual descriptors fell below 0.8 [19], it was demonstrated that the individual descriptors were highly independent of each other and were able to avoid chance correlation effects due to interdependence, for which we examined the cross-correlation matrix of the five descriptors, as shown in Table 3.

### 2.2. Model Development for Binary Mixture Compounds

In the following, five descriptors were chosen to build the mixture compound models. Regarding the generation of binary mixture descriptors, although it has been shown that descriptors are generated simply by addition [20], which is not the case, it is based on this that 11 different mixing rules (Table 1) were applied to generate the binary mixture descriptors. Not only was the choice of the hybrid rule compared in terms of the evaluation of the model quality, but also the ninth hybrid rule was more dominant in terms of the contribution of the descriptors to the molar ratio [11], and thus this hybrid rule was finally used for the construction of the hybrid model. The equation for this is

(2) $$D_{\mathrm{MIX}}\;=X_{1\;}D_{1}{}^{3}+X_{2}D_{2}{}^{3}\;$$

In addition, it will allow molecules with larger descriptor values to be more dominant in the case of large differences between the component descriptors.

Finally, the MLR for the mixture in which the equation expression is constructed based on the mixing rules and the selected descriptors is as follows:

pEC50 = 93.276 + 6.874 × [DRn-N] + 0.003 × [DHOMO] − 0.011 × [DTot-pc]-0.0000779 × [DMin-C] − 0.00001334 × [D Max-C-H](3)

N_train_ = 62, R^2^ = 0.727, RMS = 0.494, F = 159.537, Q^2^_LOO_ = 0.727, and Q^2^_pred_ = 0.725; and N_test_ = 17, R^2^ = 0.721, RMS = 0.508, F = 38.773, and q^2^_ext_ = 0.720.

When looking at the values of the statistical parameters for the internal validation, it can be demonstrated that the model has good robustness in conjunction with statistical reliability, while the external validation parameters of the model also indicate that the model has a better predictive power. Furthermore, one has a higher requirement for the model with the statistical covariates presented in Table 4. In a standard comparison of the statistical parameters (R^2^ > 0.6, q^2^_ext_ > 0.5, and k ≈ 1 (k is the slope of the regression line through the origin)), the quality of the model is in line with the requirements.

**Figure 3:**
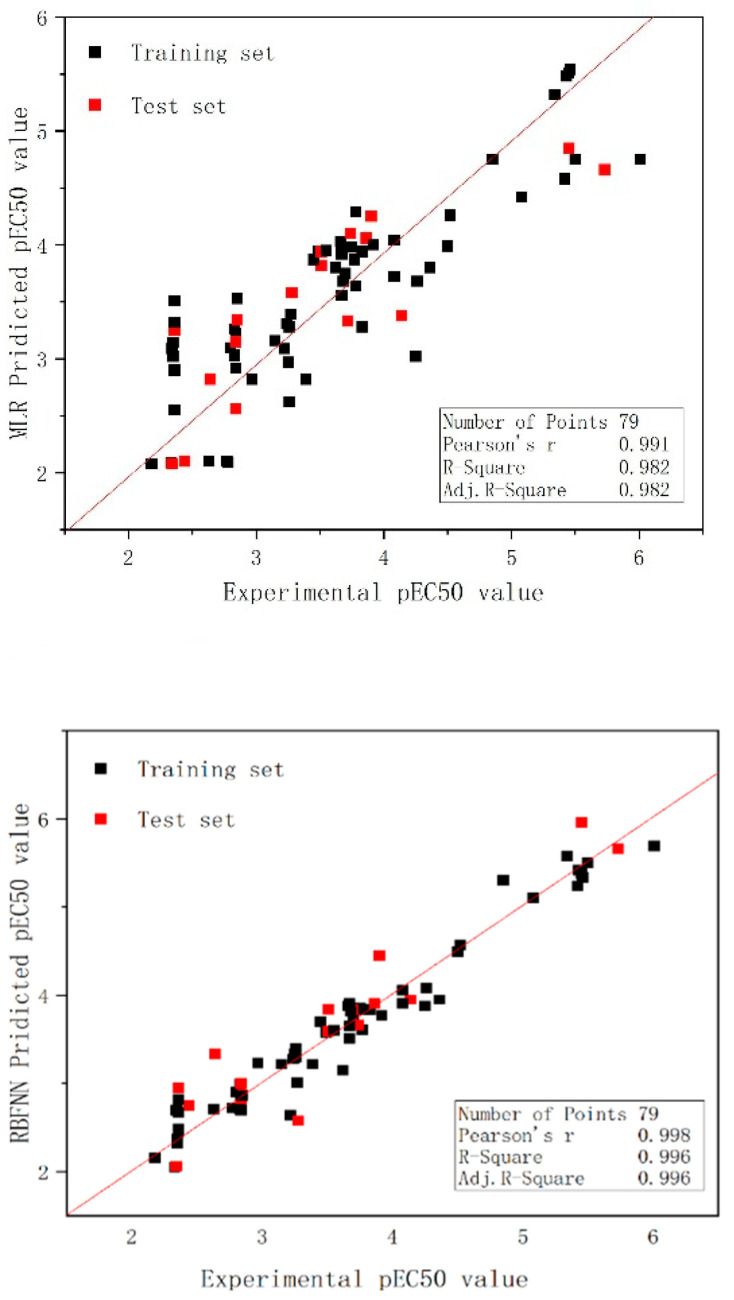
Plot of the predicted versus experimental log (EC50), including the training and test sets, from the MLR model and RBFNN model.

**Figure 4:**
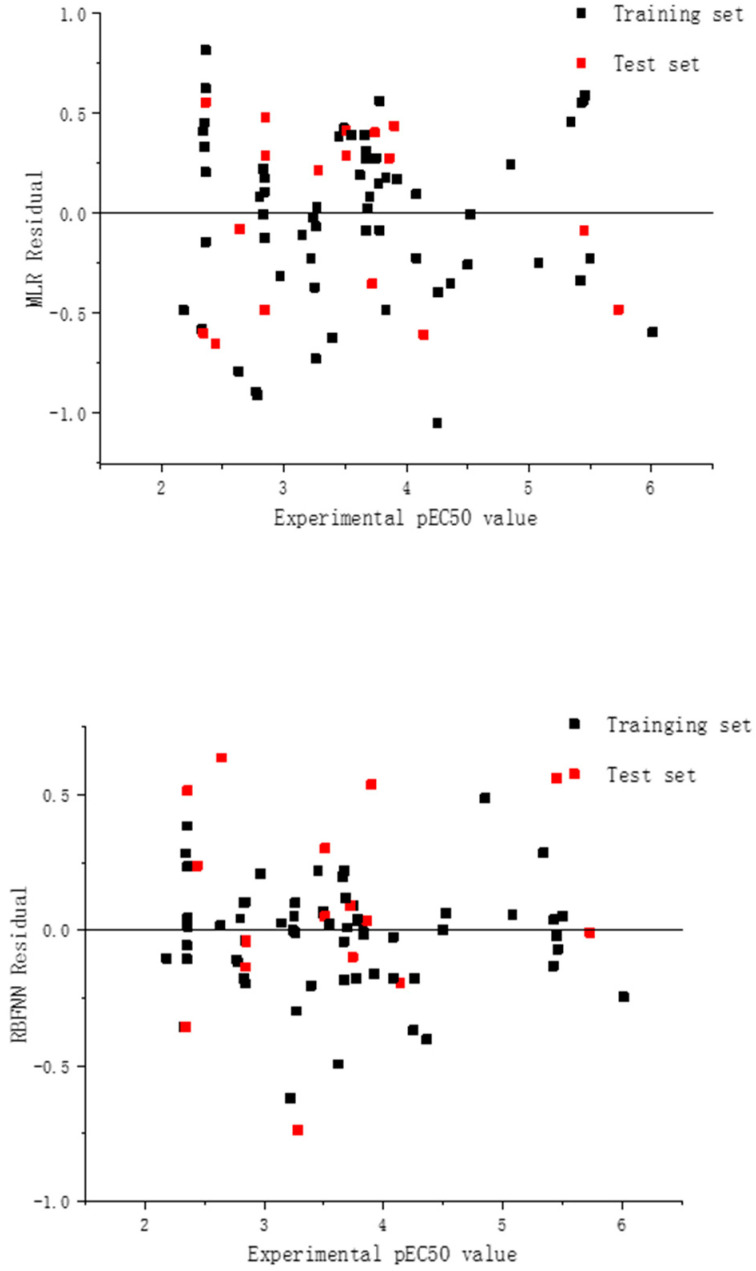
Residuals of the training and test sets by MLR and RBFNN.

The set of relevant statistics predicted by the mixtures under each of the two models is shown in Table 5; the graphs of the experimental and predicted values for the training set as well as the test set are shown in Figure 3. Moreover, Figure 4 shows the scatter plots of the residuals for all data under both models.

### 2.3. RBFNN Results Analysis

Generally, nonlinear models have outstanding predictive power compared to linear ones. In the current work, an RBFNN model was constructed using the same descriptors as those used to construct the MLR model, and the quality of the model was evaluated by randomly dividing the training set as well as the test set. In the construction of the RBFNN model, the structure of the three-layer network was constructed as 5-nk-1, denoting the number of cells in the input, hidden, and output layers. For the radial basis function (RBF), the width (r) range was controlled by starting at 0.1 in increments of 0.1 until increasing to 4. The optimal width value found for the individual compound RBFNN model was r = 4, and the optimal width value for the mixture was r = 1.6.

The prediction data of the RBFNN models for the individual compound and the binary mixture are shown in Table 1 and Table 2, respectively, and the plots of the experimental and predicted values for the training set and the test set are shown in Figure 2 and Figure 3, respectively. In addition, the scatter plots of the residuals of the two models are also shown in Figure 4. The statistical parameters for the group of the individual compounds were N_train_ = 28, R^2^ = 0.864, RMS = 0.436, F = 165.309, Q^2^_LOO_ = 0.864, and R^2^_pred_ = 0.847 for the training set, and N_test_ = 7, R^2^ = 0.941, RMS = 0.466, F = 79.300, and q^2^_ext_ = 0.834 for the test set, and it is apparent from the observations that the model exhibits superior reliability as well as predictiveness. The statistical parameters of the nonlinear model for the mixture were N^train^ = 62, R^2^ = 0.956, RMS = 0.199, F = 1279.919, Q^2^_LOO_ = 0.955, and Q^2^_pred_ = 0.855 for the training set; and N_test_ = 17, R^2^ = 0.880, RMS = 0.367, F = 110.980, and q^2^_ext_ = 0.853 for the test set. Analysis of the statistical results shows that the robustness of the model as well as its predictive power is somewhat enhanced compared to the MLR modeling. In addition, the other parameters of the model quality were calculated as shown in Table 4.

### 2.4. Validation of the Models

Y randomization tests are typically applied to confirm the degree of chance correlation of regression models. In the current work, 10 tests were conducted for each of the two models, and the amount of parameter validation for both models is shown in Table 6. As seen from the table, the low R^2^ and RMS, along with the high MAE values (see below), indicated that the chance correlation of the models will barely exist [21].

The expression for MAE is

(4) $$MAE=\frac{\sum_{i=1}^{n}\left|y^{\left(a\right)}-{\hat{y}}^{\left(b\right)}\right|}{n}\;$$

where *n* is the number of the example,$\;y^{\left(a\right)}$ is the experimental value of a single example, and ${\hat{y}}^{\left(b\right)}$ is the predicted value of a single example.

In the following, a fivefold cross-validation algorithm was applied to assess the robustness of the models built. The validation parameters, including R^2^, F, and RMS, were used to evaluate the two models, as shown in Table 7 and Table 8. According to the statistical results, it is evident that the average training quality (MLR: R^2^ = 0.727, F = 163.334, and RMS = 0.496; and RBFNN: R^2^ = 0.935, F = 881.963, and RMS = 0.237) of both models together with the average predictive quality (MLR: R^2^ = 0.737, F = 41.147, and RMS = 0.494; and RBFNN: R^2^ = 0.939, F = 233.952, and RMS = 0.243) has a good presentation, indicating that both models have relatively robust properties.

### 2.5. Model Applicability Domain Analysis

**Figure 5:**
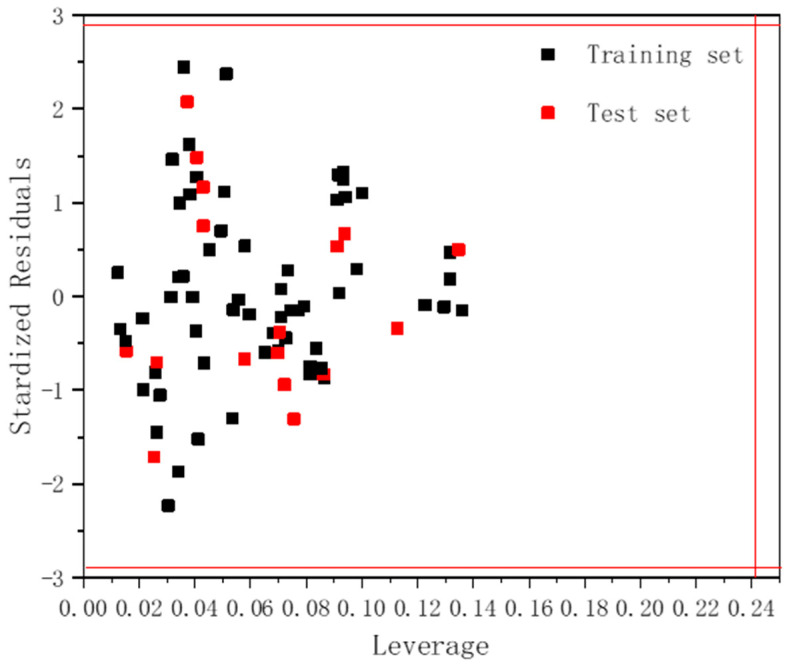
The Williams plot of the training and external test sets.

In the present study, the visual application domains of the models that can typically be observed and analyzed using Williams plots are shown in Figure 5, where the horizontal coordinate is the leverage value, the vertical coordinate is the standardized cross-validation residual, the outlier criterion (read line) for the x coordinate is set to 3 m/n (m is the number of 5 descriptors chosen and n is the number of 62 compounds used as the training set), and the outlier criterion (read line) for the y coordinate is specified as ± 3σ (σ = 0.967). In Figure 5, it can be clearly observed that both the training set compounds and the test set compounds are within the domain, indicating that a reasonably close relationship can be established between the selected descriptors and the toxicity of the compounds, and within the domain, the model is able to fit the relevant mixture toxicity predictions.

### 2.6. Discussion of Selected Descriptors

In the present work, the built model can be used to predict the compounds, including aldehydes (AHS), cyanides (CGS), sulfonamides (SAS), and methomyl (TMP). These substances contain the C, H, O, N, and S atoms. Five descriptors, namely, HOMO, Rn-N, Tot-pc, Max-C-H, and Min-C, were used to construct the QSAR model. HOMO and Rn-N have a positive effect on the increase in toxicity, and Tot-pc, Max e-n, and Min-C-H have a negative effect on the increase in toxicity. Rn-N is a constitutional descriptor, which, in the present work, mainly concerning organic molecules containing cyano and nitrogen-containing heterocycles in the compounds, is positively correlated with the increase in toxicity. HOMO, Tot-pc, Max-C-H, and Min-C are quantum-chemical descriptors; HOMO has the property of being an electron donor in a chemical reaction, and the higher the energy is, the higher the toxicity value. Tot-pc is a class of descriptors describing the polarity of a molecule, where the size of the molecular dipole depends on the distribution of the point charges. MAX-C-H describes the electron-to-charge attraction of two atoms in a C–H bond. When a bond is formed, more electronegative atoms participate in the bonding orbitals to gain some of the electrons, and the electron-to-charge attraction decreases, resulting in a decrease in electronegativity, which has a positive effect on the decrease in toxicity. Min-C is a calculation of the energy of the ground state of the C atom; the lower the energy is, the more stable and the lower the toxicity value.

## 3. Materials and Methods

### 3.1. Datasets

In the present work, 35 compounds widely present in aqueous environments, including 13 aldehydes (AHS), 11 cyanides (CGS), 10 sulfonamides (SAS), and 1 methomyl (TMP), were obtained from Mainak Chatterjee et al. [22]. In an aqueous environment, the above compounds can cause damage to the environment in the form of either individual compounds or in mixtures, which directly or indirectly have an impact on human health. The names of the individual compounds, the experimental, predicted, and residual values (pEC50) by the MLR model and the RBFNN model are presented in Table 2 (pEC50 units are in moles per liter).

Additionally, the data in Table 2 were randomly divided into 28 training sets as well as 7 test sets (marked with asterisk) to assess the performance of the individual compound models. The selection of molecular descriptors highly correlated with single toxicity in the whole dataset.

Seventy-nine binary mixtures are listed in Table 5 along with the values obtained by each of the two modeling techniques. Toxicity ratios for the binary mixtures of two individual compounds and compositional information are also listed in this table. As the QSAR studies usually did, binary mixtures of 79 species were randomly divided into 62 training sets as well as 17 test sets (marked with an asterisk) for assessing the quality of the binary mixture models.

### 3.2. Molecular Descriptors Generation and Selection

In this study, the molecular descriptors were employed as quantitative representations of the molecular structural features and were then used to build the relationship between the representative descriptors and the target of the toxicity or activity. The process is as follows: the structures of the individual compounds were first drawn by Chemdraw (PerkinElmer Informatics, Inc: Massachusetts, MA, USA) [23], followed by preliminary molecular optimization in the HyperChem 6.0 program (Hypercube, Inc., Waterloo, ON, Canada) [24] software through molecular mechanics MM+ force fields. Then, the preliminary optimized molecular structures were further optimized using the semiempirical AM1 method in the Polak-Ribière algorithm until the root mean square gradient reached 0.001 kcal/mol. Last, the molecular structures were optimized in the MOPAC 6.0 software package (Indiana University: Bloomington, IN, USA) [25], and the structure files derived from HyperChem and MOPAC were selected for structural descriptors, geometric descriptors, electrostatic descriptors, quantum chemical descriptors, and topological descriptors using the CODESSA 2.63 program (University of Florida, Gainesville, FL, USA) [26] after optimization with the same root-mean-square gradient. Furthermore, 7 of the other descriptors obtained from HyperChem (including logP) were added to the descriptor pool.

Doing this, we needed to find the representative descriptors that are more related to the toxicity of the single compounds. Thus, a heuristic method in CODESSA software was employed, which can be used to calculate a pool of relevant descriptors and subsequent determinations of the most suitable descriptors for the construction of the model.

The generalization of the descriptors for the toxicity assessment of the binary mixtures is a challenge compared to the generation of descriptors for individual compounds. Normally, the approach applied to generating mixture descriptors is the weighted descriptor generation approach [27]. Supposing that the hypothetical descriptors do not follow the simple addition, other calculation rules have been performed [11,28,29,30,31], from which the optimal mixture rule was determined based on the preliminary modeling results. For each mixing rule, the selection standard was considered in terms of the reliability and robustness of the model quality (R^2^ (correlation coefficient), R^2^_adj_ (adjusted correlation coefficient), F (Fisher test), and Q^2^_LOO_ (leave-one-out correlation coefficient)), and the expression of the equation, as shown in Table 1.

### 3.3. Model Building Technique

#### 3.3.1. Multiple Linear Regressions

As the easiest model-building statistical technique, MLR has been commonly implemented in quantitative constructive relationship models to solve regression analysis problems. It can predict the values of two or more explanatory variables from the corresponding variables, and it is essentially an extension of ordinary least squares (OLS) regression involving two and more explanatory variables as a mathematical statistical technique. Typically, multiple linear regression uses molecular descriptors as X variables to establish a mathematical relationship with the desired activity value Y (pEC50), which involves dividing the overall dataset into a test set and a training set. In a regression model, the regression coefficient bn and the intercept b_0_ of the model have the following relationship:

(5) $$Y=b_{0}+b_{1}x_{1}+b_{2}x_{2}+\cdots +b_{n}x_{n}$$

Regularly for the model, reliability and predictiveness are generally assessed using statistical parameters, including R^2^, RMS, F, Q^2^_LOO_, R^2^_pred_, q^2^_ext_, etc. For the development of the MLR model, we have chosen to do this in the CODESSA 2.63 program (University of Florida, Gainesville, FL, USA). 

#### 3.3.2. Radial Basis Function Neural Networks (RBFNN)

During the construction of the QSAR, one can consider not only the best multivariate linear model available by constructing the molecular descriptor versus the desired activity value (pEC50) but also some nonlinear models to establish the relationship, such as the RBFNN. The specifics of radial basis function neural networks have been described in several papers [32,33]. Briefly, a radial basis function neural network consists of an input layer, a hidden layer and an output layer. The input layer is virtually just an input vector and does not involve the processing of information; the hidden layer consists of k radial basis function (RBF) units; and the output layer is composed of linear neurons (LNS) [32,34]. In general, the radial basis function (RBF) serves as a Gaussian function defined by the center (Cj) and the width (Rj). The radial basis function (RBF) implements the nonlinear transformation by measuring the Euclidean distance between the input vector (X) and the center of the radial basis function (Cj):

(6) $$h_{j}=exp\left(-∥X-c_{j}∥{}^{2}/r_{j}^{2}\right)$$

(7) $$y_{k}(X)=\overset{n_{h}}{\underset{j=1}{\sum }}w_{kj}h_{j}(X)+b_{k}$$

where *y*_k_ stands for the *k*_th_ output unit of the input vector *X*, *w_kj_* for the weight relationship between the *k*_th_ output unit and the *j*_th_ implied layer unit, and *b_k_* for the respective bias.

The determination of centers and width plays a decisive role in model development. Multiple methods are used to select centers. In the current study, we chose to employ a forward subset selection routine to select the centers from the training set samples. Regarding the width selection, the width range was from 0.1 to 4, in increments of 0.1, and the best width was ultimately selected. Afterwards, the connection weights between the hidden and output layers were selected using the least squares method. For the development of the RBFNN model, we have chosen to do this in MATLAB software (Online access: https://www.mathworks.com/products/matlab.html, access on 25 November 2021).

The RBFNN model was evaluated using the same statistical parameters as the MLR model.

## 4. Conclusions

Toxicity estimation of 79 aquatic mixtures was performed by quantitative constitutive relationship modeling through MLR and RBFNN methods. Eleven different mixing rules of the hypothetical descriptors were considered to obtain the proper models. Statistical results show that the developed MLR models are more robust as well as predictive, while the RBFNN models have a better model quality compared to the former. Furthermore, the statistical results show that the developed descriptors have excellent performance for the toxicity of mixtures wirhin the applicability domain range. We conclude that the models can be effective for the toxicity prediction of aquatic contaminants and have practical value for ecological assessment.

## Figures and Tables

**Table 1:** The 11 different mixing rules and statistic parameters of the individual compound model.

NO.	Equation	R^2^	R^2^_adj_	F	Q^2^_LOO_
1	$D_{\mathrm{MIX}}\;=D_{1}+X_{2}D_{2}$	0.730	0.711	39.395	0.731
2	$D_{\mathrm{MIX}}\;={\left(X_{1\;}D_{1}+X_{2}D_{2}\right)}^{2}$	0.754	0.737	44.663	0.756
3	$D_{\mathrm{MIX}}\;=\sqrt{{\left(X_{1\;}D_{1}+X_{2}D_{2}\right)}^{2}}$	0.735	0.716	40.419	0.805
4	$D_{\mathrm{MIX}}\;=\sqrt{X_{1\;}}D_{1}$ $+\sqrt{X_{2}}D_{2}$	0.525	0.493	16.145	0.498
5	$D_{\mathrm{MIX}}\;=X_{1\;}{}^{2}D_{1}+X_{2}{}^{2}D_{2}$	0.569	0.539	19.257	0.546
6	$D_{\mathrm{MIX}}\;=X_{1\;}{}^{3}D_{1}+X_{2}{}^{3}D_{2}$	0.581	0.552	20.225	0.557
7	$D_{\mathrm{MIX}}\;=\sqrt[{3}]{X_{1\;}{}^{3}D_{1}+X_{2}{}^{3}D_{2}}$	0.561	0.531	18.681	0.541
8	$D_{\mathrm{MIX}}\;=X_{1\;}D_{1}{}^{2}+X_{2}D_{2}{}^{2}$	0.734	0.716	40.304	0.737
9	$D_{\mathrm{MIX}}\;=X_{1\;}D_{1}{}^{3}+X_{2}D_{2}{}^{3}$	0.726	0.707	38.665	0.727
10	$D_{\mathrm{MIX}}\;=\sqrt[{3}]{X_{1\;}D_{1}{}^{3}+X_{2}D_{2}{}^{3}}$	0.700	0.679	34.016	0.704
11	$D_{\mathrm{MIX}}\;=\sqrt[{2}]{X_{1\;}D_{1}{}^{2}+X_{2}D_{2}{}^{2}}$	0.715	0.696	36.689	0.719

**Table 2:** Toxicity data of the individual compounds.

Nos.	Individual Compounds	MLR			RBFNN		
		Toxicity (pEC50 (mol/L))			Toxicity (pEC50 (mol/L))		
		Experimental	Predicted	Residual	Experimental	Predicted	Residual
1	Acetaldehyde	2.36	2.74	−0.38	2.36	2.7	−0.34
2	Propionaldehyde*	2.72	2.81	−0.09	2.72	2.74	−0.02
3	Butyraldehyde	3.25	2.82	0.43	3.25	2.76	0.49
4	Valeraldehyde	3.27	2.81	0.46	3.27	2.75	0.52
5	Benzaldehyde	3.43	4.27	−0.84	3.43	4.17	−0.74
6	*p*-Nitrobenzaldehyde	4.28	4.17	0.11	4.28	4.17	0.11
7	*p*-Terephthaldehyde	4.31	4.43	−0.12	4.31	4.27	0.04
8	*p*-Chlorobenzaldehyde*	4.25	4.25	0	4.25	4	0.25
9	*p*-Bromobenzaldehyde	4.3	4.31	−0.01	4.3	4.06	0.24
10	*p*-Hydrobenzaldehyde	4.54	4.09	0.45	4.54	3.98	0.56
11	*p*-Methylbenzaldehyde	3.82	3.93	−0.11	3.82	3.93	−0.11
12	*p*-Methoxybenzaldehyde	4.03	4.41	−0.38	4.03	4.31	−0.28
13	*p*-Dimethylaminobenzaldehyde	5.4	5.02	0.38	5.4	4.75	0.65
14	Malononitrile	2.55	2.16	0.39	2.55	2.13	0.42
15	Glycolonitrile	2.98	2.87	0.11	2.98	3.01	−0.03
16	α-Hydroxyisobutyronitrile	3.61	3.39	0.22	3.61	3.74	−0.13
17	Allyl cyanide*	1.45	2.14	−0.69	1.45	2.32	−0.87
18	Benzonitrile*	3.48	3.57	−0.09	3.48	3.69	−0.21
19	Benzyl cyanide	4.23	3.18	1.05	4.23	3.09	1.14
20	Acetonitrile	0.75	0.92	−0.17	0.75	0.92	−0.17
21	Acrylonitrile	1.51	1.68	−0.17	1.51	1.69	−0.18
22	Succinonitrile	0.36	0.85	−0.49	0.36	0.91	−0.55
23	Phthalonitrile	3.51	3.83	−0.32	3.51	4.05	−0.54
24	Lactonitrile*	2.01	2.36	−0.35	2.01	2.82	−0.81
25	Sulfamethazine	4.08	4.33	−0.25	4.08	4.37	−0.29
26	Sulfapyridine	3.84	4.52	−0.68	3.84	4.57	−0.73
27	Sulfamethoxazole	4.45	4.69	−0.24	4.45	4.7	−0.25
28	Sulfadiazine	4.5	4.32	0.18	4.5	4.39	0.11
29	Sulfisoxazole	4.43	4.54	−0.11	4.43	4.57	−0.14
30	Sulfamonomethoxine	5.05	4.58	0.47	5.05	4.6	0.45
31	Sulfachloropyridazine	4.78	4.54	0.24	4.78	4.56	0.22
32	Sulfachinoxalin*	4.53	4.56	−0.03	4.53	4.58	−0.05
33	Sulfamethoxydiazine*	4.41	4.37	0.04	4.41	4.43	−0.02
34	Sulfamethoxypyridazine	4.36	4.4	−0.04	4.36	4.49	−0.13
35	Trimethoprim	3.22	3.4	−0.18	3.22	3.58	−0.36

* Test set compound.

**Table 3:** Inter-correlation between the five descriptors.

	Rn-N	HOMO	Tot-pc	Min-C	Max-C-H
Rn-N	+1.000				
HOMO	−0.297	+1.000			
Tot-pc	+0.217	+0.630	+1.000		
Min-C	−0.049	−0.622	−0.696	+1.000	
Max-C-H	−0.423	+0.311	+0.197	−0.145	+1.000

**Table 4:** The statistical results of the external test set for the MLR and RBFNNs models.

	MLR	RBFNN
R^2^	0.721	0.880
F	38.773	110.980
K	0.999	1.030
RMS q^2^_ext_	0.508 0.720	0.367 0.853

**Table 5:** The number of chemicals in the mixtures, ratio of toxic unit, experimental pEC50 mix, predicted pEC50 mix, and their corresponding residuals.

Mixture No.	Chemicals in the Mixture	The Ratio of Toxic Unit	Experimental pEC50 Mix (mol/L)	MLR		RBFNN	
				Toxicity (pEC50 (mol/L))		Toxicity (pEC50 (mol/L))	
				Predicted	Residual	Predicted	Residual
1 *	1\14	1\1	2.44	2.1	0.34	2.75	−0.31
2	2\14	1\1	2.63	2.1	0.53	2.71	−0.08
3	3\14	1\1	2.77	2.1	0.67	2.72	0.05
4	4\14	1\1	2.78	2.09	0.69	2.72	0.06
5	5\14	1\1	2.8	3.1	−0.3	2.9	−0.1
6 *	6\14	1\1	2.84	2.56	0.28	2.76	0.08
7	7\14	1\1	2.84	2.92	−0.08	2.86	−0.02
8	8\14	1\1	2.83	3.26	−0.43	2.71	0.12
9	9\14	1\1	2.84	3.22	−0.38	2.7	0.14
10 *	10\14	1\1	2.85	3.34	−0.49	2.87	−0.02
11	11\14	1\1	2.83	3.03	−0.2	2.99	−0.16
12 *	12\14	1\1	2.84	3.15	−0.31	3	−0.16
13	13\14	1\1	2.85	3.53	−0.68	2.86	−0.01
14	5\15	1\1	3.15	3.16	−0.01	3.22	−0.07
15	6\15	1\1	3.26	2.62	0.64	3.4	−0.14
16	7\15	1\1	3.25	2.97	0.28	3.34	−0.09
17	8\15	1\1	3.24	3.31	−0.07	3.28	−0.04
18	9\15	1\1	3.26	3.28	−0.02	3.29	−0.03
19	10\15	1\1	3.27	3.39	−0.12	3.01	0.26
20	11\15	1\1	3.22	3.09	0.13	2.64	0.58
21 *	13\15	1\1	3.28	3.58	−0.3	2.58	0.7
22 *	1\16	1\1	2.64	2.82	−0.18	3.34	−0.7
23	2\16	1\1	2.97	2.82	0.15	3.23	−0.26
24	3\16	1\1	3.39	2.82	0.57	3.22	0.17
25 *	5\16	1\1	3.51	3.82	−0.31	3.84	−0.33
26	6\16	1\1	3.83	3.28	0.55	3.84	−0.01
27	7\16	1\1	3.78	3.64	0.14	3.83	−0.05
28	8\16	1\1	3.75	3.98	−0.23	3.86	−0.11
29	9\16	1\1	3.83	3.94	−0.11	3.83	0
30 *	10\16	1\1	3.86	4.06	−0.2	3.91	−0.05
31	11\16	1\1	3.7	3.75	−0.05	3.73	−0.03
32	12\16	1\1	3.77	3.87	−0.1	3.61	0.16
33*	13\16	1\1	3.9	4.25	−0.35	4.45	−0.55
34	1\17	1\1	2.18	2.08	0.1	2.16	0.02
35	3\17	1\1	2.33	2.09	0.24	2.05	0.28
36 *	4\17	1\1	2.34	2.08	0.26	2.06	0.28
37	5\17	1\1	2.34	3.09	−0.75	2.7	−0.36
38	6\17	1\1	2.36	2.55	−0.19	2.48	−0.12
39	7\17	1\1	2.36	2.9	−0.54	2.67	−0.31
40 *	8\17	1\1	2.36	3.25	−0.89	2.95	−0.59
41	10\17	1\1	2.36	3.32	−0.96	2.82	−0.46
42	11\17	1\1	2.35	3.02	−0.67	2.32	0.03
43	12\17	1\1	2.35	3.14	−0.79	2.37	−0.02
44	13\17	1\1	2.36	3.51	−1.15	2.45	−0.09
45	5\18	1\1	3.45	3.87	−0.42	3.7	−0.25
46 *	6\18	1\1	3.72	3.33	0.39	3.83	−0.11
47	7\18	1\1	3.68	3.68	0	3.82	−0.14
48	8\18	1\1	3.66	4.03	−0.37	3.88	−0.22
49 *	10\18	1\1	3.74	4.1	−0.36	3.66	0.08
50	11\18	1\1	3.62	3.8	−0.18	3.15	0.47
51	12\18	1\1	3.67	3.92	−0.25	3.51	0.16
52	13\18	1\1	3.78	4.29	−0.51	3.84	−0.06
53	5\19	1\1	3.67	3.56	0.11	3.91	−0.24
54	6\19	1\1	4.25	3.02	1.23	3.88	0.37
55 *	7\19	1\1	4.14	3.38	0.76	3.95	0.19
56	8\19	1\1	4.08	3.72	0.36	4.06	0.02
57	9\19	1\1	4.26	3.68	0.58	4.08	0.18
58	10\19	1\1	4.36	3.8	0.56	3.95	0.41
59	13\19	1\1	4.5	3.99	0.51	4.49	0.01
60	25\35	1\1	5.08	4.42	0.66	5.1	−0.02
61	26\35	1\1	4.85	4.75	0.1	5.31	−0.46
62	27\35	1\1	5.5	4.75	0.75	5.5	0
63	28\35	1\1	5.42	4.58	0.84	5.24	0.18
64 *	29\35	1\1	5.45	4.85	0.6	5.96	−0.51
65	30\35	1\1	6.01	4.75	1.26	5.69	0.32
66 *	31\35	1\1	5.73	4.66	1.07	5.66	0.07
67	27\35	13396\1	3.49	3.94	−0.45	3.58	−0.09
68	27\35	8587\1	3.49	3.94	−0.45	3.58	−0.09
69	27\35	2747\1	3.49	3.94	−0.45	3.59	−0.1
70 *	27\35	858\1	3.51	3.94	−0.43	3.59	−0.08
71	27\35	274\1	3.55	3.95	−0.4	3.6	−0.05
72	27\35	85\1	3.67	3.96	−0.29	3.65	0.02
73	27\35	27\1	3.92	4	−0.08	3.77	0.15
74	27\35	15\1	4.08	4.04	0.04	3.91	0.17
75	27\35	4\1	4.52	4.26	0.26	4.57	−0.05
76	27\35	1\6	5.34	5.32	0.02	5.58	−0.24
77	27\35	1\21	5.43	5.48	−0.05	5.42	0.01
78	27\35	1\37	5.45	5.51	−0.06	5.38	0.07
79	27\35	1\116	5.46	5.54	−0.08	5.34	0.12

* Test set compounds; * A set: 1,6,11,16,21,26,31,36,41,46,51,56,61,66,71,76; * B set: 2,7,12,17,22,27,32,37,42,47,52,57,62,67,72,77; * C set: 3,8,13,18,23,28,33,38,43,48,53,58,63,68,73,78; * D set: 4,9,14,19,24,29,34,39,44,49,54,59,64,69,74,79; * D set: 5,10,15,20,25,30,35,40,45,50,55,60,65,70,75.

**Table 6:** The R^2^, RMS, and MAE values of 10 Y-randomization tests.

MLR			RBFNN		
R^2^	RMS	MAE	R^2^	RMS	MAE
0.027	1.347	1.130	0.139	1.549	1.258
0.013	1.318	1.130	0.117	1.531	1.226
0.077	1.410	1.212	0.120	1.533	1.226
0.089	1.421	1.153	0.149	1.556	1.282
0.071	1.404	1.155	0.141	1.550	1.258
0.001	1.263	1.101	0.139	1.549	1.247
0.057	1.388	1.136	0.174	1.573	1.325
0.084	1.416	1.187	0.170	1.571	1.267
0.061	1.392	1.146	0.145	1.553	1.258
0.040	1.367	1.156	0.167	1.569	1.280

**Table 7:** Validation of the MLR model.

Training Set	R^2^	F	RMS	Test Set	R^2^	F	RMS
B + C + D + T	0.711	150.419	0.506	A	0.790	52.605	0.458
A + C + D + T	0.712	150.482	0.513	B	0.799	55.744	0.426
A + B + D + T	0.723	158.899	0.506	C	0.752	42.370	0.456
A + B + C + T	0.743	176.638	0.478	D	0.674	28.902	0.563
A + B + C + D	0.744	180.233	0.479	T	0.674	26.113	0.566
Average	0.727	163.334	0.496		0.737	41.147	0.494

**Table 8:** Validation of the RBFNN model.

Training Set	R^2^	F	RMS	Test Set	R^2^	F	RMS
B + C + D + T	0.937	912.852	0.237	A	0.931	187.779	0.277
A + C + D + T	0.932	837.216	0.212	B	0.956	306.656	0.216
A + B + D + T	0.933	845.700	0.252	C	0.944	235.582	0.218
A + B+ C + T	0.929	799.833	0.254	D	0.958	317.511	0.207
A + B+ C + D	0.942	1014.217	0.231	T	0.904	122.236	0.298
Average	0.935	881.963	0.237		0.939	233.952	0.243

## Supplementary Materials

The following supporting information can be downloaded at: https://www.mdpi.com/article/10.3390/molecules27196389/s1. The total and the eliminated descriptor.
