# Supplementary material for: Toxicity Assessment of the Binary Mixtures of Aquatic Organisms Based on Different Hypothetical Descriptors
Source: Molecules. 2022 Sep 27;27(19):6389. doi: 10.3390/molecules27196389 (PMC9571779; doi:10.3390/molecules27196389)
Supplement: Supplementary file 1 [file molecules-27-06389-s001.zip › molecules-1886970-supplementary.pdf]

Toxicity assessment of the binary mixtures of aquatic organism based on different hypothetical descriptors

Meng Ji [1], Lihong Zhang [1], Xuming Zhuang [1], Chunyuan Tian[1], Feng Luan [1*] and M. Natália D. S. Cordeiro [2]

[1] College of Chemistry and Chemical Engineering, Yantai University, Yantai 264005, China
[2] LAQV@REQUIMTE/Department of Chemistry and Biochemistry, Faculty of Sciences, University of Porto, 4169-007 Porto, Portugal
* Corresponding author. E-mail address: fluan@sina.com (F. Luan).

The total and the eliminated descriptor in this study:

Using heuristic method
total 614 descriptors selected
calculating 1-parameter correlations
descriptor "Relative number of C atoms"
descriptor "Number of F atoms"
descriptor "Relative number of F atoms"
descriptor "Number of Cl atoms"
descriptor "Relative number of Cl atoms".
descriptor "Number of Br atoms".
descriptor "Relative number of Br atoms".
descriptor "Number of I atoms"
descriptor "Relative number of I atoms"
descriptor "Number of P atoms"
descriptor "Relative number of P atoms"
descriptor "Kier flexibility index"
descriptor "Average Information content (order 0)"
descriptor "Average Structural Information content (order 1)"
descriptor "Average Bonding Information content (order 1)"
descriptor "Average Complementary Information content (order 2)"
descriptor "Average Bonding Information content (order 2)"
descriptor "Balaban index"
descriptor "YZ Shadow / YZ Rectangle"
descriptor "ZX Shadow / ZX Rectangle"
descriptor "Min partial charge for a C atom [Zefirov's PC]"
descriptor "Max partial charge for a O atom [Zefirov's PC]"
descriptor "Min partial charge for a O atom [Zefirov's PC]"
descriptor "Min partial charge for a H atom [Zefirov's PC]"
descriptor "Polarity parameter / square distance"
descriptor "FPSA-1 Fractional PPSA (PPSA-1/TMSA) [Zefirov's PC]"
descriptor "FNSA-1 Fractional PNSA (PNSA-1/TMSA) [Zefirov's PC]"
descriptor "FNSA-3 Fractional PNSA (PNSA-3/TMSA) [Zefirov's PC]"

descriptor "HDSA H-donors surface area [Zefirov's PC]"
descriptor "FHDSA Fractional HDSA (HDSA/TMSA) [Zefirov's PC]"
descriptor "HASA H-acceptors surface area [Zefirov's PC]"
descriptor "FHASA Fractional HASA (HASA/TMSA) [Zefirov's PC]"
descriptor "HBSA H-bonding surface area [Zefirov's PC]"
descriptor "FHBSA Fractional HBSA (HBSA/TMSA) [Zefirov's PC]"
descriptor "HDCA H-donors charged surface area [Zefirov's PC]"
descriptor "FHDCA Fractional HDCA (HDCA/TMSA) [Zefirov's PC]"
descriptor "HACA H-acceptors charged surface area [Zefirov's PC]"
descriptor "FHACA Fractional HACA (HACA/TMSA) [Zefirov's PC]"
descriptor "HBCA H-bonding charged surface area [Zefirov's PC]"
descriptor "FHBCA Fractional HBSA (HBSA/TMSA) [Zefirov's PC]"
descriptor "count of H-donors sites [Zefirov's PC]"
descriptor "HA dependent HDSA-1/TMSA [Zefirov's PC]"
descriptor "HA dependent HDSA-2/TMSA [Zefirov's PC]"
descriptor "HA dependent HDSA-2/SQRT(TMSA) [Zefirov's PC]"
descriptor "HA dependent HDCA-1/TMSA [Zefirov's PC]"
descriptor "HASA-2 [Zefirov's PC]"
descriptor "HACA-1 [Zefirov's PC]"
descriptor "HACA-2 [Zefirov's PC]"
descriptor "HACA-2/SQRT(TMSA) [Zefirov's PC]"
descriptor "Min nucleoph. react. index for a O atom"
descriptor "Max nucleoph. react. index for a O atom"
descriptor "Avg nucleoph. react. index for a O atom"
descriptor "Min electroph. react. index for a O atom"
descriptor "Max electroph. react. index for a O atom"
descriptor "Avg electroph. react. index for a O atom"
descriptor "Max 1-electron react. index for a C atom"
descriptor "Min 1-electron react. index for a O atom"
descriptor "Max 1-electron react. index for a O atom"
descriptor "Avg 1-electron react. index for a O atom"
descriptor "Max net atomic charge for a O atom"
descriptor "Min net atomic charge for a O atom"
descriptor "Image of the Onsager-Kirkwood solvation energy"
descriptor "RPCG Relative positive charge (QMPOS/QTPLUS) [Quantum-Chemical PC]"
descriptor "count of H-donors sites [Quantum-Chemical PC]"
descriptor "HA dependent HDSA-2/TMSA [Quantum-Chemical PC]"
descriptor "HA dependent HDSA-2/SQRT(TMSA) [Quantum-Chemical PC]"
descriptor "HA dependent HDCA-1/TMSA [Quantum-Chemical PC]"
descriptor "HA dependent HDCA-2/TMSA [Quantum-Chemical PC]"
descriptor "HASA-2 [Quantum-Chemical PC]"
descriptor "HACA-1/TMSA [Quantum-Chemical PC]"
descriptor "Max SIGMA-SIGMA bond order"
descriptor "Min valency of a O atom"

descriptor "Max valency of a O atom"
descriptor "Avg valency of a O atom"
descriptor "Min (>0.1) bond order of a O atom"
descriptor "Max bond order of a O atom"
descriptor "Avg bond order of a O atom"
descriptor "Max bond order of a H atom"
descriptor "Max atomic state energy for a C atom"
descriptor "Min e-e repulsion for a O atom"
descriptor "Max e-e repulsion for a O atom"
descriptor "Min e-n attraction for a O atom"
descriptor "Max e-n attraction for a O atom"
descriptor "Min atomic state energy for a O atom"
descriptor "Max atomic state energy for a O atom"
descriptor "Min e-n attraction for a C-C bond"
descriptor "Min n-n repulsion for a C-C bond"
descriptor "Min coulombic interaction for a C-C bond"
descriptor "Min resonance energy for a C-O bond"
descriptor "Max resonance energy for a C-O bond"
descriptor "Min exchange energy for a C-O bond"
descriptor "Max exchange energy for a C-O bond"
descriptor "Min e-e repulsion for a C-O bond"
descriptor "Max e-e repulsion for a C-O bond"
descriptor "Min e-n attraction for a C-O bond"
descriptor "Max e-n attraction for a C-O bond"
descriptor "Min n-n repulsion for a C-O bond"
descriptor "Max n-n repulsion for a C-O bond"
descriptor "Min coulombic interaction for a C-O bond"
descriptor "Max coulombic interaction for a C-O bond"
descriptor "Min total interaction for a C-O bond"
descriptor "Max total interaction for a C-O bond"
descriptor "Max e-e repulsion for a C-H bond"
descriptor "Min n-n repulsion for a C-H bond"
descriptor "Min coulombic interaction for a C-H bond"
descriptor "Min total interaction for a C-H bond"
descriptor "Tot molecular 2-center exchange energy / # of atoms"
descriptor "Kier shape index (order 3)"
descriptor "Max partial charge for a N atom [Zefirov's PC]"
descriptor "Min partial charge for a N atom [Zefirov's PC]"
descriptor "Min nucleoph. react. index for a N atom"
descriptor "Max nucleoph. react. index for a N atom"
descriptor "Avg nucleoph. react. index for a N atom"
descriptor "Min electroph. react. index for a N atom"
descriptor "Max electroph. react. index for a N atom"
descriptor "Avg electroph. react. index for a N atom"

descriptor "Min 1-electron react. index for a N atom"
descriptor "Max 1-electron react. index for a N atom"
descriptor "Avg 1-electron react. index for a N atom"
descriptor "Max net atomic charge for a N atom"
descriptor "Min net atomic charge for a N atom"
descriptor "Min valency of a N atom"
descriptor "Max valency of a N atom"
descriptor "Avg valency of a N atom"
descriptor "Min (>0.1) bond order of a N atom"
descriptor "Max bond order of a N atom"
descriptor "Avg bond order of a N atom"
descriptor "Min e-e repulsion for a N atom"
descriptor "Max e-e repulsion for a N atom"
descriptor "Min e-n attraction for a N atom"
descriptor "Max e-n attraction for a N atom"
descriptor "Min atomic state energy for a N atom"
descriptor "Max atomic state energy for a N atom"
descriptor "Min resonance energy for a C-N bond"
descriptor "Max resonance energy for a C-N bond"
descriptor "Min exchange energy for a C-N bond"
descriptor "Max exchange energy for a C-N bond"
descriptor "Min e-e repulsion for a C-N bond"
descriptor "Max e-e repulsion for a C-N bond"
descriptor "Min e-n attraction for a C-N bond"
descriptor "Max e-n attraction for a C-N bond"
descriptor "Min n-n repulsion for a C-N bond"
descriptor "Max n-n repulsion for a C-N bond"
descriptor "Min coulombic interaction for a C-N bond"
descriptor "Max coulombic interaction for a C-N bond"
descriptor "Min total interaction for a C-N bond"
descriptor "Max total interaction for a C-N bond"
descriptor "Min resonance energy for a N-O bond"
descriptor "Max resonance energy for a N-O bond"
descriptor "Min exchange energy for a N-O bond"
descriptor "Max exchange energy for a N-O bond"
descriptor "Min e-e repulsion for a N-O bond"
descriptor "Max e-e repulsion for a N-O bond"
descriptor "Min e-n attraction for a N-O bond"
descriptor "Max e-n attraction for a N-O bond"
descriptor "Min n-n repulsion for a N-O bond"
descriptor "Max n-n repulsion for a N-O bond"
descriptor "Min coulombic interaction for a N-O bond"
descriptor "Max coulombic interaction for a N-O bond"
descriptor "Min total interaction for a N-O bond"

descriptor "Max total interaction for a N-O bond"
descriptor "Max partial charge for a Cl atom [Zefirov's PC]"
descriptor "Min partial charge for a Cl atom [Zefirov's PC]"
descriptor "Min nucleoph. react. index for a Cl atom"
descriptor "Max nucleoph. react. index for a Cl atom"
descriptor "Avg nucleoph. react. index for a Cl atom"
descriptor "Min electroph. react. index for a Cl atom"
descriptor "Max electroph. react. index for a Cl atom"
descriptor "Avg electroph. react. index for a Cl atom"
descriptor "Min 1-electron react. index for a Cl atom"
descriptor "Max 1-electron react. index for a Cl atom"
descriptor "Avg 1-electron react. index for a Cl atom" has missing values, skipped
descriptor "Max net atomic charge for a Cl atom"
descriptor "Min net atomic charge for a Cl atom"
descriptor "Min valency of a Cl atom"
descriptor "Max valency of a Cl atom"
descriptor "Avg valency of a Cl atom"
descriptor "Min (>0.1) bond order of a Cl atom"
descriptor "Max bond order of a Cl atom"
descriptor "Avg bond order of a Cl atom"
descriptor "Min e-e repulsion for a Cl atom"
descriptor "Max e-e repulsion for a Cl atom"
descriptor "Min e-n attraction for a Cl atom"
descriptor "Max e-n attraction for a Cl atom"
descriptor "Min atomic state energy for a Cl atom"
descriptor "Max atomic state energy for a Cl atom"
descriptor "Min resonance energy for a C-Cl bond"
descriptor "Max resonance energy for a C-Cl bond"
descriptor "Min exchange energy for a C-Cl bond"
descriptor "Max exchange energy for a C-Cl bond"
descriptor "Min e-e repulsion for a C-Cl bond"
descriptor "Max e-e repulsion for a C-Cl bond"
descriptor "Min e-n attraction for a C-Cl bond"
descriptor "Max e-n attraction for a C-Cl bond"
descriptor "Min n-n repulsion for a C-Cl bond"
descriptor "Max n-n repulsion for a C-Cl bond"
descriptor "Min coulombic interaction for a C-Cl bond"
descriptor "Max coulombic interaction for a C-Cl bond"
descriptor "Min total interaction for a C-Cl bond"
descriptor "Max total interaction for a C-Cl bond"
descriptor "Max partial charge for a Br atom [Zefirov's PC]"
descriptor "Min partial charge for a Br atom [Zefirov's PC]"
descriptor "Min nucleoph. react. index for a Br atom"
descriptor "Max nucleoph. react. index for a Br atom"

descriptor "Avg nucleoph. react. index for a Br atom"
descriptor "Min electroph. react. index for a Br atom"
descriptor "Max electroph. react. index for a Br atom"
descriptor "Avg electroph. react. index for a Br atom"
descriptor "Min 1-electron react. index for a Br atom"
descriptor "Max 1-electron react. index for a Br atom"
descriptor "Avg 1-electron react. index for a Br atom"
descriptor "Max net atomic charge for a Br atom"
descriptor "Min net atomic charge for a Br atom"
descriptor "Min valency of a Br atom"
descriptor "Max valency of a Br atom"
descriptor "Avg valency of a Br atom"
descriptor "Min (>0.1) bond order of a Br atom"
descriptor "Max bond order of a Br atom"
descriptor "Avg bond order of a Br atom"
descriptor "Min e-e repulsion for a Br atom"
descriptor "Max e-e repulsion for a Br atom"
descriptor "Min e-n attraction for a Br atom"
descriptor "Max e-n attraction for a Br atom"
descriptor "Min atomic state energy for a Br atom"
descriptor "Max atomic state energy for a Br atom"
descriptor "Min resonance energy for a Br-C bond"
descriptor "Max resonance energy for a Br-C bond"
descriptor "Min exchange energy for a Br-C bond"
descriptor "Max exchange energy for a Br-C bond"
descriptor "Min e-e repulsion for a Br-C bond"
descriptor "Max e-e repulsion for a Br-C bond"
descriptor "Min e-n attraction for a Br-C bond"
descriptor "Max e-n attraction for a Br-C bond"
descriptor "Min n-n repulsion for a Br-C bond"
descriptor "Max n-n repulsion for a Br-C bond"
descriptor "Min coulombic interaction for a Br-C bond"
descriptor "Max coulombic interaction for a Br-C bond"
descriptor "Min total interaction for a Br-C bond"
descriptor "Max total interaction for a Br-C bond"
descriptor "Min resonance energy for a H-O bond"
descriptor "Max resonance energy for a H-O bond"
descriptor "Min exchange energy for a H-O bond"
descriptor "Max exchange energy for a H-O bond"
descriptor "Min e-e repulsion for a H-O bond"
descriptor "Max e-e repulsion for a H-O bond"
descriptor "Min e-n attraction for a H-O bond"
descriptor "Max e-n attraction for a H-O bond"
descriptor "Min n-n repulsion for a H-O bond"

descriptor "Max n-n repulsion for a H-O bond"
descriptor "Min coulombic interaction for a H-O bond"
descriptor "Max coulombic interaction for a H-O bond"
descriptor "Min total interaction for a H-O bond"
descriptor "Max total interaction for a H-O bond"
descriptor "Max partial charge for a S atom [Zefirov's PC]"
descriptor "Min partial charge for a S atom [Zefirov's PC]"
descriptor "Min nucleoph. react. index for a S atom"
descriptor "Max nucleoph. react. index for a S atom"
descriptor "Avg nucleoph. react. index for a S atom"
descriptor "Min electroph. react. index for a S atom"
descriptor "Max electroph. react. index for a S atom"
descriptor "Avg electroph. react. index for a S atom"
descriptor "Min 1-electron react. index for a S atom"
descriptor "Max 1-electron react. index for a S atom"
descriptor "Avg 1-electron react. index for a S atom"
descriptor "Max net atomic charge for a S atom"
descriptor "Min net atomic charge for a S atom"
descriptor "Min valency of a S atom"
descriptor "Max valency of a S atom"
descriptor "Avg valency of a S atom"
descriptor "Min (>0.1) bond order of a S atom"
descriptor "Max bond order of a S atom"
descriptor "Avg bond order of a S atom"
descriptor "Min e-e repulsion for a S atom"
descriptor "Max e-e repulsion for a S atom"
descriptor "Min e-n attraction for a S atom"
descriptor "Max e-n attraction for a S atom"
descriptor "Min atomic state energy for a S atom"
descriptor "Max atomic state energy for a S atom"
descriptor "Min resonance energy for a C-S bond"
descriptor "Max resonance energy for a C-S bond"
descriptor "Min exchange energy for a C-S bond"
descriptor "Max exchange energy for a C-S bond"
descriptor "Min e-e repulsion for a C-S bond"
descriptor "Max e-e repulsion for a C-S bond"
descriptor "Min e-n attraction for a C-S bond"
descriptor "Max e-n attraction for a C-S bond"
descriptor "Min n-n repulsion for a C-S bond"
descriptor "Max n-n repulsion for a C-S bond"
descriptor "Min coulombic interaction for a C-S bond"
descriptor "Max coulombic interaction for a C-S bond"
descriptor "Min total interaction for a C-S bond"
descriptor "Max total interaction for a C-S bond"

descriptor "Min resonance energy for a O-S bond"
descriptor "Max resonance energy for a O-S bond"
descriptor "Min exchange energy for a O-S bond"
descriptor "Max exchange energy for a O-S bond"
descriptor "Min e-e repulsion for a O-S bond"
descriptor "Max e-e repulsion for a O-S bond"
descriptor "Min e-n attraction for a O-S bond"
descriptor "Max e-n attraction for a O-S bond"
descriptor "Min n-n repulsion for a O-S bond"
descriptor "Max n-n repulsion for a O-S bond"
descriptor "Min coulombic interaction for a O-S bond"
descriptor "Max coulombic interaction for a O-S bond"
descriptor "Min total interaction for a O-S bond"
descriptor "Max total interaction for a O-S bond"
descriptor "Min resonance energy for a N-S bond"
descriptor "Max resonance energy for a N-S bond"
descriptor "Min exchange energy for a N-S bond"
descriptor "Max exchange energy for a N-S bond"
descriptor "Min e-e repulsion for a N-S bond"
descriptor "Max e-e repulsion for a N-S bond"
descriptor "Min e-n attraction for a N-S bond"
descriptor "Max e-n attraction for a N-S bond"
descriptor "Min n-n repulsion for a N-S bond"
descriptor "Max n-n repulsion for a N-S bond"
descriptor "Min coulombic interaction for a N-S bond"
descriptor "Max coulombic interaction for a N-S bond"
descriptor "Min total interaction for a N-S bond"
descriptor "Max total interaction for a N-S bond"
descriptor "Min resonance energy for a H-N bond"
descriptor "Max resonance energy for a H-N bond"
descriptor "Min exchange energy for a H-N bond"
descriptor "Max exchange energy for a H-N bond"
descriptor "Min e-e repulsion for a H-N bond"
descriptor "Max e-e repulsion for a H-N bond"
descriptor "Min e-n attraction for a H-N bond"
descriptor "Max e-n attraction for a H-N bond"
descriptor "Min n-n repulsion for a H-N bond"
descriptor "Max n-n repulsion for a H-N bond"
descriptor "Min coulombic interaction for a H-N bond"
descriptor "Max coulombic interaction for a H-N bond"
descriptor "Min total interaction for a H-N bond"
descriptor "Max total interaction for a H-N bond"
descriptor "Min resonance energy for a N-N bond"
descriptor "Max resonance energy for a N-N bond"

descriptor "Min exchange energy for a N-N bond"
descriptor "Max exchange energy for a N-N bond"
descriptor "Min e-e repulsion for a N-N bond"
descriptor "Max e-e repulsion for a N-N bond"
descriptor "Min e-n attraction for a N-N bond"
descriptor "Max e-n attraction for a N-N bond"
descriptor "Min n-n repulsion for a N-N bond"
descriptor "Max n-n repulsion for a N-N bond"
descriptor "Min coulombic interaction for a N-N bond"
descriptor "Max coulombic interaction for a N-N bond"
descriptor "Min total interaction for a N-N bond"
descriptor "Max total interaction for a N-N bond"
removed 348 descriptors
total 266 descriptors left
descriptor "Min e-e repulsion for a C atom"
descriptor "Max net atomic charge for a C atom"
descriptor "Number of benzene rings"
descriptor "Moment of inertia A"
descriptor "Principal moment of inertia A / # of atoms"
descriptor "Molecular surface area"
descriptor "Moment of inertia C"
descriptor "Principal moment of inertia B"
descriptor "Moment of inertia B"
descriptor "Principal moment of inertia B / # of atoms"
descriptor "Average Structural Information content (order 0)"
descriptor "HASA-1/TMSA [Quantum-Chemical PC]"
descriptor "Randic index (order 3)"
descriptor "descriptor 6"
descriptor "TMSA Total molecular surface area [Zefirov's PC]"
descriptor "TMSA Total molecular surface area [Quantum-Chemical PC]"
descriptor "ALFA polarizability (DIP)"
descriptor "Molecular volume"
descriptor "Randic index (order 1)"
descriptor "Kier&Hall index (order 0)"
descriptor "descriptor 3"
descriptor "descriptor 2"
descriptor "Randic index (order 0)"
descriptor "Max PI-PI bond order"
descriptor "(1/6)X GAMMA polarizability (DIP)"
descriptor "Kier&Hall index (order 1)"
descriptor "Kier&Hall index (order 3)"
descriptor "No. of occupied electronic levels"
descriptor "Tot molecular 1-center E-N attraction"
descriptor "Tot molecular 1-center E-E repulsion"

descriptor "Gravitation index (all bonds)"
descriptor "Gravitation index (all pairs)"
descriptor "Molecular weight"
descriptor "Relative number of aromatic bonds"
descriptor "Number of bonds"
descriptor "Tot molecular 2-center resonance energy"
descriptor "Number of atoms"
descriptor "Bonding Information content (order 2)"
descriptor "Structural Information content (order 1)"
descriptor "Bonding Information content (order 1)"
descriptor "Information content (order 2)"
descriptor "Information content (order 1)"
descriptor "1X BETA polarizability (DIP)"
descriptor "WNSA-2 Weighted PNSA (PNSA2*TMSA/1000) [Zefirov's PC]"
descriptor "WPSA-3 Weighted PPSA (PPSA3*TMSA/1000) [Quantum-Chemical PC]"
descriptor "HASA-2/TMSA [Quantum-Chemical PC]"
descriptor "Max partial charge (Qmax)"
descriptor "PNSA-3 Atomic charge weighted PNSA [Quantum-Chemical PC]"
descriptor "WNSA-3 Weighted PNSA (PNSA3*TMSA/1000) [Quantum-Chemical PC]"
descriptor "Max net atomic charge for a H atom"
descriptor "Min e-n attraction for a H atom"
descriptor "Min atomic state energy for a H atom"
descriptor "Min valency of a H atom"
descriptor "PNSA-2 Total charge weighted PNSA [Quantum-Chemical PC]"
descriptor "Structural Information content (order 0)"
descriptor "Bonding Information content (order 0)"
descriptor "DPSA-2 Difference in CPSAs (PPSA2-PNSA2) [Quantum-Chemical PC]"
descriptor "PPSA-2 Total charge weighted PPSA [Quantum-Chemical PC]"
descriptor "Max exchange energy for a C-C bond"
descriptor "Max resonance energy for a C-C bond"
descriptor "Topographic electronic index (all bonds) [Zefirov's PC]"
descriptor "DPSA-2 Difference in CPSAs (PPSA2-PNSA2) [Zefirov's PC]"
descriptor "HASA-1 [Quantum-Chemical PC]"
descriptor "Max e-e repulsion for a C atom"
descriptor "Max e-n attraction for a C atom"
descriptor "Number of S   atoms"
descriptor "Relative number of S   atoms"
descriptor "HACA H-acceptors charged surface area [Quantum-Chemical PC]"
descriptor "FHBCA Fractional HBSA (HBSA/TMSA) [Quantum-Chemical PC]"
descriptor "HA dependent HDCA-1 [Zefirov's PC]"
descriptor "HASA-2/SQRT(TMSA) [Quantum-Chemical PC]"
descriptor "FPSA-1 Fractional PPSA (PPSA-1/TMSA) [Quantum-Chemical PC]"
descriptor "PPSA-2 Total charge weighted PPSA [Zefirov's PC]"
descriptor "HASA H-acceptors surface area [Quantum-Chemical PC]"

descriptor "FHDCA Fractional HDCA (HDCA/TMSA) [Quantum-Chemical PC]"
descriptor "HDSA H-donors surface area [Quantum-Chemical PC]"
descriptor "Tot molecular 1-center E-N attraction / # of atoms"
descriptor "count of H-acceptor sites [Zefirov's PC]"
descriptor "HA dependent HDSA-2 [Zefirov's PC]"
descriptor "HA dependent HDCA-1 [Quantum-Chemical PC]"
descriptor "HA dependent HDSA-2 [Quantum-Chemical PC]"
descriptor "Max e-n attraction for a H atom"
descriptor "Min net atomic charge for a H atom"
descriptor "Max atomic state energy for a H atom"
descriptor "Max valency of a H atom"
removed 85 highly correlated descriptors
total 181 descriptors left
1: HOMO - LUMO energy gap
2: Min atomic state energy for a C atom
3: Min e-n attraction for a C atom
4: Min nucleoph. react. index for a C atom
5: HOMO energy
6: Number of aromatic bonds
7: Relative number of rings
8: HOMO-1 energy
9: - Average Complementary Information content (order 0)
10: - Principal moment of inertia A
11: - Max coulombic interaction for a C-H bond
12: - Max resonance energy for a C-H bond
13: - Relative number of triple bonds
14: - Max total interaction for a C-H bond
15: - Min (>0.1) bond order of a H atom
16: - XY Shadow
17: - Average Information content (order 2)
18: - Max n-n repulsion for a C-H bond
19: - Principal moment of inertia C
20: Number of rings
21: Principal moment of inertia C / # of atoms
22: - Average Bonding Information content (order 0)
23: - Number of C atoms
24: - RNCS Relative negative charged SA (SAMNEG*RNCG) [Zefirov's PC]
25: - Tot point-charge comp. of the molecular dipole
26: - Min atomic orbital electronic population
27: - HASA-1/TMSA [Zefirov's PC]
28: - LUMO energy
29: - Avg electroph. react. index for a C atom
30: 12 - descriptor 5
31: - Max partial charge for a C atom [Zefirov's PC]

32: - FNSA-2 Fractional PNSA (PNSA-2/TMSA) [Zefirov's PC]
33: - Avg bond order of a C atom
34: - Max bond order of a C atom
35: - 1X GAMMA polarizability (DIP)
36: - WNSA-3 Weighted PNSA (PNSA3*TMSA/1000) [Zefirov's PC]
37: - Average Information content (order 1)
38: - Kier&Hall index (order 2)
39: - Max atomic orbital electronic population
40: - Randic index (order 2)
41: - WNSA-1 Weighted PNSA (PNSA1*TMSA/1000) [Zefirov's PC]
42: - descriptor 7
43: - Number of O atoms
44: - Relative number of benzene rings
45: - RPCS Relative positive charged SA (SAMPOS*RPCG) [Zefirov's PC]
46: - Number of triple bonds
47: - Tot molecular 2-center exchange energy
48: - DPSA-3 Difference in CPSAs (PPSA3-PNSA3) [Zefirov's PC]
49: - descriptor 1
50: - Tot dipole of the molecule
51: - Max antibonding contribution of a MO
52: - FPSA-3 Fractional PPSA (PPSA-3/TMSA) [Quantum-Chemical PC]
53: - PPSA-1 Partial positive surface area [Quantum-Chemical PC]
54: - Structural Information content (order 2)
55: - (1/2) X BETA polarizability (DIP)
56: - PNSA-2 Total charge weighted PNSA [Zefirov's PC]
57: - RNCG Relative negative charge (QMNEG/QTMINUS) [Quantum-Chemical PC]
58: - PPSA-3 Atomic charge weighted PPSA [Quantum-Chemical PC]
59: - PNSA-3 Atomic charge weighted PNSA [Zefirov's PC]
60: - Tot molecular electrostatic interaction
61: - HASA-2/TMSA [Zefirov's PC]
62: - LUMO+1 energy
63: - Complementary Information content (order 0)
64: - HACA-2 [Quantum-Chemical PC]
65: - Max partial charge for a H atom [Zefirov's PC]
66: - Kier shape index (order 1)
67: - Min (>0.1) bond order of a C atom
68: - DPSA-3 Difference in CPSAs (PPSA3-PNSA3) [Quantum-Chemical PC]
69: - Min valency of a C atom
70: - Min e-e repulsion for a H atom
71: - Polarity parameter (Qmax-Qmin)
72: - WNSA-1 Weighted PNSA (PNSA1*TMSA/1000) [Quantum-Chemical PC]
73: - Complementary Information content (order 1)
74: - FNSA-3 Fractional PNSA (PNSA-3/TMSA) [Quantum-Chemical PC]
75: - RNCG Relative negative charge (QMNEG/QTMINUS) [Zefirov's PC]

76: - FNSA-2 Fractional PNSA (PNSA-2/TMSA) [Quantum-Chemical PC]
77: - HACA-2/SQRT(TMSA) [Quantum-Chemical PC]
78: - PNSA-1 Partial negative surface area [Zefirov's PC]
79: - WPSA-1 Weighted PPSA (PPSA1*TMSA/1000) [Quantum-Chemical PC]
80: - Information content (order 0)
81: - Min e-e repulsion for a C-H bond
82: - ZX Shadow
83: - Avg 1-electron react. index for a C atom
84: - FPSA-2 Fractional PPSA (PPSA-2/TMSA) [Quantum-Chemical PC]
85: - Number of H atoms
86: - Min net atomic charge
87: - Number of double bonds
88: - Min partial charge (Qmin)
89: - YZ Shadow
90: - PPSA-3 Atomic charge weighted PPSA [Zefirov's PC]
91: - Min e-n attraction for a C-H bond
92: - Max total interaction for a C-C bond
93: - WNSA-2 Weighted PNSA (PNSA2*TMSA/1000) [Quantum-Chemical PC]
94: - PPSA-1 Partial positive surface area [Zefirov's PC]
95: - RPCS Relative positive charged SA (SAMPOS*RPCG) [Quantum-Chemical PC]
96: - Max e-n attraction for a C-C bond
97: - RPCG Relative positive charge (QMPOS/QTPLUS) [Zefirov's PC]
98: - Topographic electronic index (all pairs) [Zefirov's PC]
99: - HASA-1 [Zefirov's PC]
100: - Max e-e repulsion for a C-C bond
101: - WPSA-1 Weighted PPSA (PPSA1*TMSA/1000) [Zefirov's PC]
102: - Number of single bonds
103: - Max SIGMA-PI bond order
104: - WPSA-3 Weighted PPSA (PPSA3*TMSA/1000) [Zefirov's PC]
105: - WPSA-2 Weighted PPSA (PPSA2*TMSA/1000) [Quantum-Chemical PC]
106: - Max net atomic charge
107: - Min net atomic charge for a C atom
108: - FHACA Fractional HACA (HACA/TMSA) [Quantum-Chemical PC]
109: - Relative number of O atoms
110: - DPSA-1 Difference in CPSAs (PPSA1-PNSA1) [Quantum-Chemical PC]
111: - HA dependent HDCA-2 [Zefirov's PC]
112: - Wiener index
113: - Avg bond order of a H atom
114: - HBCA H-bonding charged surface area [Quantum-Chemical PC]
115: - Avg nucleoph. react. index for a C atom
116: - Avg valency of a H atom
117: - Complementary Information content (order 2)
118: - HASA-2/SQRT(TMSA) [Zefirov's PC]
119: - FNSA-1 Fractional PNSA (PNSA-1/TMSA) [Quantum-Chemical PC]

120: - HACA-1/TMSA [Zefirov's PC]
121: - Min electroph. react. index for a C atom
122: - FPSA-2 Fractional PPSA (PPSA-2/TMSA) [Zefirov's PC]
123: - Tot hybridization comp. of the molecular dipole
124: - Relative number of single bonds
125: - HBSA H-bonding surface area [Quantum-Chemical PC]
126: - Max n-n repulsion for a C-C bond
127: - Max electroph. react. index for a C atom
128: - HDCA H-donors charged surface area [Quantum-Chemical PC]
129: - HACA-2/TMSA [Quantum-Chemical PC]
130: - HACA-1 [Quantum-Chemical PC]
131: - FHDSA Fractional HDSA (HDSA/TMSA) [Quantum-Chemical PC]
132: - Min e-e repulsion for a C-C bond
133: - Tot molecular 1-center E-E repulsion / # of atoms
134: - RNCS Relative negative charged SA (SAMNEG*RNCG) [Quantum-Chemical PC]
135: - HA dependent HDCA-2/SQRT(TMSA) [Zefirov's PC]
136: - Average Complementary Information content (order 1)
137: - Max coulombic interaction for a C-C bond
138 - Final heat of formation / # of atoms
139: - Min 1-electron react. index for a C atom
140: - Relative number of double bonds
141: - FHBSA Fractional HBSA (HBSA/TMSA) [Quantum-Chemical PC]
142: - descriptor 4
143: - Relative number of N atoms
144: - count of H-acceptor sites [Quantum-Chemical PC]
145: - Min total interaction for a C-C bond
146: - min (#HA, #HD) [Zefirov's PC]
147: - FHASA Fractional HASA (HASA/TMSA) [Quantum-Chemical PC]
148: - Kier shape index (order 2)
149: - Avg valency of a C atom
150: - WPSA-2 Weighted PPSA (PPSA2*TMSA/1000) [Zefirov's PC]
151: - Max exchange energy for a C-H bond
152: - Relative molecular weight
153: - Max bonding contribution of a MO
154: - HA dependent HDCA-2 [Quantum-Chemical PC]
155: - min(#HA, #HD) [Quantum-Chemical PC]
156: - FPSA-3 Fractional PPSA (PPSA-3/TMSA) [Zefirov's PC]
157: - Average Structural Information content (order 2)
158: - XY Shadow / XY Rectangle
159: - Max nucleoph. react. index for a C atom
160: - Final heat of formation
161: - Min exchange energy for a C-C bond
162: - PNSA-1 Partial negative surface area [Quantum-Chemical PC]
163: - Molecular volume / XYZ Box

164: - No. of occupied electronic levels / # of atoms
165: - Max valency of a C atom
166: - Tot molecular 2-center resonance energy / # of atoms
167: - HA dependent HDSA-1 [Zefirov's PC]
168: - HACA-2/TMSA [Zefirov's PC]
169: - HA dependent HDCA-2/TMSA [Zefirov's PC]
170: - Min resonance energy for a C-C bond
171: - Max e-e repulsion for a H atom
172: - Min resonance energy for a C-H bond
173: - HA dependent HDSA-1 [Quantum-Chemical PC]
174: - Min exchange energy for a C-H bond
175: - HA dependent HDCA-2/SQRT(TMSA) [Quantum-Chemical PC]
176: - Number of N atoms
177: - HA dependent HDSA-1/TMSA [Quantum-Chemical PC]
178: - Relative number of H atoms
179: - Max e-n attraction for a C-H bond
180: - DPSA-1 Difference in CPSAs (PPSA1-PNSA1) [Zefirov's PC]
181: - Tot molecular electrostatic interaction / # of atoms
calculating 2-parameter correlations...
best 2-parameter correlations:
1: R^2=0.7564 F= 38.8060
2: R^2=0.7480 F= 37.0979
3: R^2=0.7470 F= 36.9167
4: R^2=0.7310 F= 33.9729
5: R^2=0.7307 F= 33.9103
6: R^2=0.7283 F= 33.5141
7: R^2=0.7275 F= 33.3749
8: R^2=0.7253 F= 33.0056
9: R^2=0.7241 F= 32.8031
10: R^2=0.7177 F= 31.7854
descriptors involved:
345 - Min atomic state energy for a C atom
60 - Relative number of N atoms
389 - Min exchange energy for a C-H bond
251 - Tot point-charge comp. of the molecular dipole
363 - Min e-e repulsion for a C-C bond
237 - Min 1-electron react. index for a C atom
343 - Min e-n attraction for a C atom
372 - Max total interaction for a C-C bond
280 - RNCS Relative negative charged SA (SAMNEG*RNCG) [Quantum-Chemical PC]
368 - Max n-n repulsion for a C-C bond
370 - Max coulombic interaction for a C-C bond
209 - HASA-2/SQRT(TMSA) [Zefirov's PC]
394 - Max e-n attraction for a C-H bond

calculating multi-parameter correlations...
best correlations (R^2):

1: R^2=0.8869 F= 34.5062 (5 descriptors) 345 60 251 394 221
2: R^2=0.8849 F= 33.8327 (5 descriptors) 345 60 251 394 119
3: R^2=0.8844 F= 33.6557 (5 descriptors) 345 60 251 394 258
4: R^2=0.8843 F= 33.6444 (5 descriptors) 345 60 251 394 415
5: R^2=0.8817 F= 32.8060 (5 descriptors) 345 389 388 327 251
6: R^2=0.8809 F= 32.5548 (5 descriptors) 345 60 251 394 411
7: R^2=0.8797 F= 32.1838 (5 descriptors) 345 60 251 390 415
8: R^2=0.8797 F= 32.1830 (5 descriptors) 345 389 388 327 253
9: R^2=0.8792 F= 32.0253 (5 descriptors) 345 60 251 394 225
10: R^2=0.8789 F= 31.9197 (5 descriptors) 345 60 251 390 237
